# GLP1-RA and SGLT2-i: Implementation and Insulin Deescalation Strategies

**DOI:** 10.1007/s10557-025-07744-8

**Published:** 2025-07-10

**Authors:** Arthi Palani, Sagar Patel, Shriya Patel, Shivani Srivastava, Jay Patel, Salman Salehin, Yochai Birnbaum, Joseph Allencherril

**Affiliations:** 1 Department of Medicine, Rutgers New Jersey Medical School, Newark, NJ USA; 2 Division of Cardiology, University of Texas Medical Branch, Galveston, TX USA; 3 Section of Cardiology, Baylor College of Medicine, Houston, TX USA

**Keywords:** Diabetes, Cardiovascular disease, SGLT2-i, GLP-1RA, Heart failure, Outcomes, Insulin de-escalation

## Abstract

**Purpose of Review:**

This review will summarize the most contemporary trials for glucagon-like peptide 1 receptor agonists (GLP1-RA) and sodium-glucose cotransporter 2 inhibitors (SGLT2-i) and present guidance on management and recommendations on adjunctive tapering of insulin in patients with insulin-dependent diabetes mellitus, which we term “de-insulinization.”

**Recent Findings:**

GLP1-RA and SGLT2-i are the principal classes of diabetes medications evidencing cardiovascular benefit. However, there is limited consensus on how to effectively prescribe and maintain these medications for patients on other glucose-lowering therapy, especially insulin.

**Summary:**

Patients with type 2 diabetes and cardiovascular disease should be started on either a GLP-1RA, SGLT-i, or both in high-risk cases, while simultaneously tapering any prescribed insulin regimen. Initial selection and transition of therapy should be approached individually and systematically, with prioritization of patients with other comorbidities such as coronary artery disease, chronic kidney disease, and other diabetes complications.

## Introduction

Cardiovascular disease (CVD) remains the leading cause of death in the USA, accounting for 928,741 deaths in the year 2024. Coronary artery disease (CAD) led at 40.3% of the deaths attributable to CVD, followed by stroke, other minor CVD causes, high blood pressure, heart failure, and peripheral arterial disease [1]. Among other comorbidities, diabetes is a major cause of cardiovascular morbidity and mortality in the USA. Rates of CVD deaths, both in men and women, are 1.7 times higher among adults with diabetes than adults diagnosed without diabetes due to increased risk of stroke and myocardial infarction [2].

Over the last decade, several studies have investigated the role of glucose-lowering drugs in cardiovascular outcomes, especially glucagon-like peptide-1 receptor agonists (GLP1-RA) and sodium-glucose cotransporter 2 inhibitors (SGLT2i) which have demonstrated substantial improvements in major cardiovascular events, kidney function outcomes, and hospitalizations [3]. These agents are recommended as first-line therapy by the American Diabetes Association and the European Society of Cardiology for patients with diabetes and underlying cardiovascular disease [4, 5]. Despite these recommendations, there is still limited guidance on how to initiate these agents in patients already on insulin and consequently tapering insulin regimens.

## GLP-1 Receptor Agonists

GLP-1RA have been found to have several effects in various organs and tissues. Primarily, these medications decrease postprandial glucagon secretion, slow gastric emptying, and promote appetite suppression as a treatment for type 2 diabetes and obesity. In addition, GLP-1 agents have also been shown to have several cardiovascular benefits with blood pressure control, atherosclerosis, heart failure, and cardiovascular disease outcomes. Physiological GLP-1 receptors have also been found in atrial and ventricular cardiomyocytes, suggesting a role in blood pressure reduction, improvement of myocardial function, and increased natriuresis [3, 6]. Beyond these effects, GLP-1 receptor agonists influence cardiovascular physiology through both peripheral and central mechanisms. They have been shown to reduce sympathetic outflow, enhance myocardial glucose uptake, and improve left ventricular function [7]. In a randomized control trial, patients who received exenatide were studied 6 months after ST-segment elevation MI (STEMI) after undergoing balloon angioplasty and stent placement. These patients were found to have a smaller infarct size on cardiac MRI and improvements in diastolic function and global longitudinal strain on repeat echocardiography [8].

Currently, there are seven different approved GLP-1RAs on the market: liraglutide once daily, semaglutide once weekly, exenatide extended-release (ER) once weekly, dulaglutide once weekly, oral semaglutide once daily, exenatide twice daily, and lixisenatide once daily. The short-acting formulations help reduce postprandial glucose levels, while the long-acting agents affect both fasting and prandial glucose levels [6]. Exenatide and lixisenatide are based on the peptide exendin-4, while the rest are GLP-1-based agents. Exendin-4 is a peptide that shares 53% sequence homology with human GLP-1 and can bind and activate GLP-1 receptors with similar potency to GLP-1RAs. These molecules are also naturally resistant to the degradation of dipeptidyl peptidase-4 (DDP-4) enzymes. The GLP-1-based GLP-1RAs closely mimic the naturally produced GLP-1 hormone in the human body, with > 90% amino acid homology [9]. This may play a role in the improved outcomes seen with these agents versus exendin-4-based agents.

Nine trials have been published investigating the cardiovascular outcome benefits of GLP-1RA, as listed in Tables 1 and 2. Of note, albiglutide from the HARMONY trial was discontinued in 2017 due to modest sales. The primary outcome studied across all the trials was the first occurrence of death from cardiovascular causes, nonfatal myocardial infarction, or nonfatal stroke. LEADER (Liraglutide Effect and Action in Diabetes: Evaluation of Cardiovascular Outcomes Results) trial revealed that patients who received liraglutide had a lower risk of the primary composite outcome as listed above, cardiovascular death, death from any cause, and microvascular events than the placebo group [10]. The lower risk of the primary end point in LEADER was primarily driven by a significantly lower rate of cardiovascular death. SUSTAIN-6 (Semaglutide in Subjects with Type 2 Diabetes) was the next trial published, lending credence to the cardiovascular benefits of semaglutide. This trial showed that patients who received semaglutide had a 26% lower risk of the primary composite outcome than the placebo group, principally driven by a significant 39% reduction in nonfatal stroke, with no significant difference in the rate of cardiovascular death. The risk reductions noted were similar across both the 0.5 mg and 1 mg weekly groups of patients [11].

**Table 1:** Baseline characteristics of the patients enrolled in GLP-1RA trials

Trial	LEADER (7)	SUSTAIN 6 (8)	EXSCEL (9)	Harmony Outcomes ([12])	REWIND (12)	PIONEER 6 (13)	Amplitude O (14)	SELECT (15)	SUMMIT (16)
Number of patients	9440	3297	14,752	9463	9901	3183	4076	17,604	693
Age, mean	64.3	64.6	62	64.2	66.2	66	64.5	61.6	65.3
Male sex (%)	6033 (63.6)	1025 (31.1)	9149 (62.0)	6569 (69.4)	5311 (53.6)	2176 (68.4)	2732 (67.0)	12,732 (72.3)	338 (48.8)
Ischemic heart disease no. (%)	3536 (37.5)	991 (30.1)	7794 (52.8)	6678 (70.6)	3114 (31.5)	2695 (84.7)	3650 (89.5)	14,452 (82.1)	217 (31.3)
Glycated hemoglobin %, mean	8.7	8.7	-	8.7	7.4	8.2	8.9	5.8	-
BMI, kg/m^2^, mean	32.5	32.8	31.8	32.3	32.3	32.3	32.7	33.3	38.3
NYHA HF Class II–III no. (%)	1305 (13.8)	386 (11.7)	2389 (16.2)	1922 (20.3)	853 (8.6)	-	737 (18.1)	4286 (24.3)	731(100)
Chronic kidney disease* no. (%)	1934 (20.5)	1026 (31.1)	3177 (21.5)	-	2199 (22.2)	856 (26.9)	-	-	-

^*^Estimated glomerular filtration rate < 60 mL/min

**Table 2:** Summary of primary composite outcomes for GLP-1RA trials

Trial	Year	Study Population	GLP-1RA	Dose	Route	Follow-Up	GLP-1RA *Outcome*	Placebo *Outcome*	HR (95% CI)	Primary Outcome
LEADER (7)	2016	type 2 diabetes and at least one cardiovascular coexisting condition or cardiovascular risk factor*	liraglutide	1.8 mg daily	subcutaneous	3.8 years	608 (13.0)	694 (14.9)	0.87 (0.78–0.97)	composite of death from cardiovascular causes, nonfatal myocardial infarction, or nonfatal stroke
SUSTAIN 6 (8)	2017	type 2 diabetes and at least one cardiovascular coexisting condition or cardiovascular risk factor*	semaglutide	0.5 mg or 1 mg weekly	subcutaneous	5 weeks	108 (6.6)	146 (8.9)	0.74 (0.58–0.95)	composite of death from cardiovascular causes, nonfatal myocardial infarction, or nonfatal stroke
EXSCEL (9)	2017	type 2 diabetes, approximately 70% had previous cardiovascular events	exenatide	2 mg weekly	subcutaneous	3.2 years	839 (11.4)	905 (12.2)	0.91 (0.83–1.00)	composite of death from cardiovascular causes, nonfatal myocardial infarction, or nonfatal stroke
Harmony Outcomes (11)	2018	type 2 diabetes and established coronary disease, cerebrovascular or peripheral arterial disease	albiglutide	30-50 mg weekly	subcutaneous	1.5 years	338 (7)	238 (9)	0.78 (0.68–0.90)	composite of death from cardiovascular causes, nonfatal myocardial infarction, or nonfatal stroke
REWIND (12)	2019	type 2 diabetes with established vascular disease**	dulaglutide	1.5 mg weekly	subcutaneous	5.4 years	594 (12)	663 (13.4)	0.88 (0.79–0.99)	composite of death from cardiovascular causes, nonfatal myocardial infarction, or nonfatal stroke
Amplitude O (14)	2021	type 2 diabetes and history of cardiovascular disease, or kidney disease with at least one additional cardiovascular risk factor	efpeglenatide	4 or 6 mg weekly	subcutaneous	1.8 years	189 (7.0)	125 (9.2)	0.73 (0.58–0.92)	composite of death from cardiovascular causes, nonfatal myocardial infarction, or nonfatal stroke
PIONEER 6 (13)	2019	established cardiovascular disease or chronic kidney disease	semaglutide	14 mg daily	oral	15 months	61 (3.8)	76 (4.8)	0.79 (0.57–1.11)	composite of death from cardiovascular causes, nonfatal myocardial infarction, or nonfatal stroke
SELECT (15)	2023	age of 45 or more with BMI of 27 or greater and established cardiovascular disease	semaglutide	2.4 mg weekly	subcutaneous	3.3 years	569 (6.5)	701 (8.0)	0.80 (0.72–0.90)	composite of death from cardiovascular causes, nonfatal myocardial infarction, or nonfatal stroke
SUMMIT (16)	2024	age of 40 or more with chronic heart failure, ejection fraction of at least 50% and BMI of at least 30	tirzepatide	15 mg weekly	subcutaneous	2 years	36 (9.9)	56 (15.3)	0.62 (0.41–0.95)	death from cardiovascular causes or a worsening heart-failure event

^*^Coexisting cardiovascular conditions: coronary heart disease, cerebrovascular disease, peripheral vascular disease, chronic kidney disease of stage 3 or greater, or chronic heart failure of New York Heart Association class II or III. Risk factors as determined by the investigator: microalbuminuria or proteinuria, hypertension and left ventricular hypertrophy, left ventricular systolic or diastolic dysfunction, or an ankle–brachial index of less than 0.9

^**^Previous myocardial infarction, ischemic stroke, revascularization, hospital admission for unstable angina, or imaging evidence of myocardial ischemia); those aged 55 years or older had to have myocardial ischemia, coronary, carotid, or lower extremity artery stenosis exceeding 50%, left ventricular hypertrophy, estimated glomerular filtration rate (eGFR) less than 60 mL/min per 1·73 m^2^, or albuminuria; and those aged 60 years or older had to have at least two of tobacco use, dyslipidemia, hypertension, or abdominal obesity

EXSCEL (Effects of Once-Weekly Exenatide on Cardiovascular Outcomes in Type 2 Diabetes) revealed that the incidence of major cardiovascular events was not significantly different in the group receiving exenatide compared to placebo. This was thought to be due to several factors. Compared to the LEADER trial, the follow-up time in EXSCEL was much shorter (3.2 years vs. 3.8 years) as well as the duration of therapy (2.5 years vs. 3.5 years) and rate of treatment discontinuation [13]. The HARMONY trial did not demonstrate any mortality benefit but showed a significant reduction in myocardial infarction in the treatment group with albiglutide vs. placebo [14]. REWIND (Researching Cardiovascular Events with a Weekly Incretin in Diabetes) investigated the use of once weekly dulaglutide on cardiovascular events in patients with type 2 diabetes, revealing a significant reduction in the primary composite outcome [15]. PIONEER-6 (Peptide Innovation for Early Diabetes Treatment) demonstrated the similar cardiovascular benefit of oral semaglutide to that of the subcutaneous form seen in SUSTAIN-6 [16]. Amplitude O (Effect of Efpeglenatide on Cardiovascular Outcomes) revealed that patients with type 2 DM and CVD or kidney disease receiving once weekly efpeglenatide had a 27% lower incidence of the primary composite outcome and 32% lower risk of a composite renal outcome event compared to those receiving placebo [17]. Efpeglenatide has not yet been approved by the U.S. Food and Drug Administration (FDA) at this time.

SELECT (Semaglutide Effects on Cardiovascular Outcomes in People with Overweight or Obesity) investigated the role of semaglutide in reducing cardiovascular outcomes in patients with obesity and pre-existing cardiovascular disease, but no diabetes was investigated. In this trial, the use of semaglutide at a dose of 2.4 mg weekly was found to be superior to placebo in reducing the incidence of death from cardiovascular causes, nonfatal myocardial infarction, or nonfatal stroke [18].

Although the cardioprotective effects of GLP-1RA are more established in atherosclerotic cardiovascular disease than in heart failure, recent studies demonstrate a modest but clinically meaningful benefit in heart failure with preserved ejection fraction (HFpEF), particularly among obese patients. STEP-HF (semaglutide in patients with heart failure with preserved ejection fraction and obesity) revealed a large reduction in symptoms and physical limitations in patients who received semaglutide 2.4 mg using the Kansas City Cardiomyopathy Questionnaire Clinical Summary Score (KCCQ-CSS). Those in the treatment group also had greater improvements in exercise function and greater weight loss than placebo [19]. SUMMIT was the most recent trial investigating cardiovascular outcomes of tirzepatide in patients with obesity and heart failure with preserved ejection fraction (HFpEF). Tirzepatide is a GLP-1RA that also acts on glucose-dependent insulinotropic polypeptide (GIP) receptors. Death from cardiovascular causes or worsening heart failure events occurred less in patients treated with up to 15 mg tirzepatide weekly compared to placebo [20].

In addition to their cardiovascular impact, GLP-1RAs have demonstrated renal protective effects in clinical trials. In the LEADER trial, liraglutide reduced the risk of new-onset persistent macroalbuminuria, doubling of serum creatinine, and need for renal replacement therapy by 22% compared to placebo [10]. Similarly, in the AWARD-7 trial, dulaglutide was shown to preserve eGFR more effectively than insulin glargine over 52 weeks in patients with moderate-to-severe CKD, despite similar glycemic control [21]. These renal benefits are thought to be mediated by mechanisms including reduced glomerular hyperfiltration, enhanced natriuresis, and systemic anti-inflammatory effects. Together, these findings expand the clinical utility of GLP-1RAs to include kidney protection and symptom improvement in HFpEF, beyond their role in reducing major adverse cardiovascular events (Table 3).

**Table 3:** Baseline characteristics of patients enrolled in SGLT-2 inhibitor trials

Trial	EMPA-REG OUTCOME (19)	CANVAS (23)	DECLARE-TIMI 58 (25)	CREDENCE (24)	DAPA-HF (26)	VERTIS-CV (28)	DAPA-CKD (27)	EMPA-kidney (20)	EMPEROR-preserved (21)	EMPEROR-reduced (22)
Number of patients	7020	10,142	17,160	4401	4744	8246	4304	6609	5988	3730
Age, mean	63.2	63.3	63.9	63.0	66.3	64.4	61.9	63.9	71.9	66.9
Male sex (%)	5081 (72.4)	6509 (64.2)	10,738 (62.6)	2907 (66.1)	3635 (76.6)	5769 (69.9)	2879 (66.9)	2192 (33.2)	3312 (55.3)	2837 (76.1)
BMI kg/m^2^, mean	30.7	32.0	32.1	31.3	28.2	31.9	29.5	29.8	29.8	27.9
Ischemic heart disease (%)	5308 (75.6)	5721 (56.4)	5658 (33.0)	1313 (29.8)	2674 (56.3)	6256 (75.9)	1610 (37.4)	1765 (26.7)	2117 (35.4)	1929 (51.7)
NYHA HF Class II–III no. (%)	706 (10.1)	1461 (14.4)	1724 (10.0)	652 (14.8)	4744 (100)	1958 (23.7)	468 (10.9)	-	5984 (99.9)	3730 (100)
Chronic kidney disease* no. (%)	1819 (25.9)	-	-	1266 (28.8)	1926 (40.6)	1807 (21.9)	1328 (30.9)	6609 (100)	2988 (49.9)	1799 (48.2)
Diabetes mellitus (%)	7020 (100)	10,142 (100)	17,160 (100)	4401 (100)	1983 (41.8)	8246 (100)	2906 (67.5)	3040 (46.0)	2938 (49.1)	1856 (49.8)
Glycated hemoglobin %, mean	8.1	8.2	8.3	8.3	-	8.2	-	-	-	-

^*^Estimated glomerular filtration rate < 60 mL/min

### SGLT2 Inhibitors

There are several cardiovascular outcome trials testing the efficacy of SGLT2-i as listed in Table 3 and Table 4. The specific outcomes evaluated across each of the studies can be grouped into the three major adverse cardiovascular events (MACE): cardiovascular death, nonfatal MI, and nonfatal stroke, progression of kidney disease, and heart failure hospitalizations. EMPA-REG OUTCOME (Empagliflozin Cardiovascular Outcome Event Trial in Type 2 Diabetes Mellitus Patients) was the first major trial published evaluating SGLT2-i use for reduction of MACE in patients with type 2 diabetes and known CVD on empagliflozin. Both doses of 10 mg and 25 mg had similar hazard ratios for cardiovascular outcomes; thus, the choice of the dose will likely depend on metabolic targets [22].

**Table 4:** Summary of primary outcomes for SGLT-2 inhibitor Trials

Trial	Year	Study Population	Drug	Dose	SGLT-2 *Outcome*	Placebo *Outcome*	HR (95%CI)	Follow-up	Primary Outcome
EMPA-REG OUTCOME (19)	2015	type 2 diabetes and established cardiovascular disease	empagliflozin	10 or 25 mg daily	10.5%	12.1%	0.86 (0.74–0.99)	3.1 years	composite of death from cardiovascular causes, nonfatal myocardial infarction, or nonfatal stroke
CANVAS (23)	2017	type 2 diabetes and symptomatic atherosclerotic cardiovascular disease	canagliflozin	100 or 300 mg daily	26.9*	31.5*	0.86 (0.75–0.97)	3.6 years	composite of death from cardiovascular causes, nonfatal myocardial infarction, or nonfatal stroke
DECLARE-TIMI 58 (25)	2019	type 2 diabetes and either established cardiovascular disease or multiple risk factors	dapagliflozin	10 mg daily	8.8%	9.4%	0.93 (0.84–1.03)	4.2 years	composite of death from cardiovascular causes, nonfatal myocardial infarction, or nonfatal stroke
CREDENCE (24)	2019	type 2 diabetes, chronic kidney disease and albuminuria	canagliflozin	100 mg daily	43.2*	61.2*	0.70 (0.59–0.82)	2.6 years	composite of end-stage kidney disease (dialysis, transplantation, or a sustained estimated GFR of < 15 ml per minute per 1.73 m^2^), a doubling of the serum creatinine level, or death from renal or cardiovascular causes
VERTIS-CV (28)	2020	type 2 diabetes and established atherosclerotic cardiovascular disease	ertugliflozin	5 or 15 mg daily	11.9%	11.9%	0.97 (0.85–1.11)	3.5 years	composite of death from cardiovascular causes, nonfatal myocardial infarction, or nonfatal stroke
DAPA-CKD (27)	2020	chronic kidney disease and albuminuria, with or without diabetes	dapagliflozin	10 mg daily	9.2%	14.5%	0.61 (0.51–0.72)	2.4 years	composite of a sustained decline in the estimated GFR of at least 50%, end-stage kidney disease, or death from renal or cardiovascular causes
EMPA-Kidney (20)	2023	chronic kidney disease, with or without diabetes	empagliflozin	10 mg daily	13.1%	16.9%	0.72 (0.64–0.82)	2 years	composite of progression of kidney disease (defined as end-stage kidney disease, a sustained decrease in eGFR to < 10 ml per minute per 1.73 m^2^, a sustained decrease in eGFR of ≥ 40% from baseline, or death from renal causes) or death from cardiovascular causes
DAPA-HF (26)	2019	ejection fraction of 40% or less, and New York Heart Association (NYHA) class II-IV symptoms	dapagliflozin	10 mg daily	16.3%	21.2%	0.74 (0.65–0.85)	1.5 years	composite of cardiovascular death or hospitalization for worsening heart failure
EMPEROR-Preserved (21)	2021	ejection fraction of 40% or greater and NYHA class II-IV symptoms	empagliflozin	10 mg daily	13.8%	17.1%	0.79 (0.69–0.90)	2.2 years	composite of cardiovascular death or hospitalization for worsening heart failure
EMPEROR–Reduced (22)	2020	ejection fraction of 40% or less, and NYHA class II-IV symptoms	empagliflozin	10 mg daily	19.4%	24.7%	0.75 (0.65–0.86)	1.3 years	composite of cardiovascular death or hospitalization for worsening heart failure

The CANVAS (Canagliflozin and Cardiovascular and Renal Events in Type 2 Diabetes) trial revealed lower risk for death from cardiovascular causes, nonfatal myocardial infarction, and nonfatal stroke in patients with established type 2 diabetes and cardiovascular disease treated with canagliflozin in comparison to placebo. Of note, there was also an increased rate of amputation noted for unknown mechanisms [23]. DECLARE-TIMI 58 (Dapagliflozin and Cardiovascular Outcomes in Type 2 Diabetes) assessed cardiovascular outcomes associated with dapagliflozin 10 mg daily. Unlike the aforementioned, a lower rate of MACE than placebo was not observed. However, a lower rate of cardiovascular death and heart failure hospitalizations was seen [24]. VERTIS-CV (Cardiovascular outcomes with Ertugliflozin in Type 2 Diabetes) was the only study to assess outcomes associated with ertugliflozin at 5 mg and 15 mg. Among the patients who received ertugliflozin, a MACE occurred in 11.9%, which was the same as the placebo group. Ertugliflozin was found to be non-inferior to placebo with respect to major cardiovascular events. The patients who received 5 mg were found to have − 0.70% in HbA1c level versus − 0.72% in the group that received 15 mg [25].

Two landmark trials for heart failure management and SGLTI2-i were EMPA-REDUCED (Empagliflozin Outcome Trial in Patients with Chronic Heart Failure and a Reduced Ejection Fraction) and EMPA-PRESERVED (Empagliflozin Outcome Trial in Patient with Chronic Heart Failure with Preserved Ejection Fraction). These trials revealed that empagliflozin-treated patients with left ventricular ejection fraction < 40% and > 40% experienced a lower risk of cardiovascular death or hospitalization for heart failure regardless of the presence or absence of diabetes [26, 27]. DAPA-HF (Dapagliflozin and Prevention of Adverse Outcomes in Heart Failure) revealed that the use of dapagliflozin in patients with New York Heart Association (NYHA) class II, III, or IV heart failure and an ejection fraction of 40% resulted in a lower risk of worsening heart failure and cardiovascular death. It was also shown to be effective in patients without type 2 diabetes, supporting the therapeutic effects of SGLT2-i beyond diabetes [28].

EMPA-KIDNEY (Empagliflozin in Patients with Chronic Kidney Disease) showed that in patients with chronic kidney disease, empagliflozin leads to a lower risk of progression of kidney disease [29]. The CREDENCE trial (Canagliflozin and Renal Events in Diabetes with Established Nephropathy Clinical Evaluation) also evaluated the cardiovascular risk reduction of canagliflozin, but in patients with type 2 diabetes and established chronic kidney disease. The resulting lower risk of end-stage renal disease (ESRD), renal insufficiency, or death from renal and cardiovascular causes in this subset indicated that canagliflozin may be an effective treatment for cardiovascular and renal protection [30]. The DAPA-CKD (Dapagliflozin and Prevention of Adverse Outcomes in Chronic Kidney Disease) trial specifically included patients with chronic kidney disease with or without diabetes to receive dapagliflozin and found that these patients experienced a lower risk of ESRD, GFR decline of at least 50%, or death from renal or cardiovascular causes in comparison to placebo [31].

## Practical Prescription Strategies

The use of GLP-1 RA and SGLT-2 inhibitors has been proven to show cardiovascular risk reduction, unlike insulin use. These agents have several practical benefits for patients including promotion of weight loss, fewer needlesticks, ease of administration, and lower risk of hypoglycemia. Patients with diabetes and clinical atherosclerotic cardiovascular disease may preferentially be started on one or more of these contemporary agents. Combination therapy of GLP-1RA and insulin has been exclusively studied in several trials. Overall, this combination has been shown to improve glycemic control and reduce body weight with low risk of hypoglycemia. When initiating GLP-1RA for patients already on insulin, clinicians should be cautious about insulin titration. Studies in which insulin was titrated aggressively to obtain glycemic control resulted in less weight loss. In contrast, protocols focused on insulin sparing tended to show more weight loss but modest glycemic benefits [32].

One observational study conducted in the UK showed a 0.51% reduction in HbA1c and a 5.8-kg reduction in weight, with one in six patients discontinuing insulin after 26 weeks of exenatide-insulin combination therapy. Adding liraglutide to insulin therapy has also been seen to have significant reductions in HbA1c and weight loss, a 0.8% reduction of HbA1c and a mean weight loss of 5.1 kg, respectively. There is heterogeneity in data regarding insulin dose adjustments after initiating GLP-1 RA therapy. Some studies report an increase in insulin dose in patient populations on oral diabetic agents, although this was found in the context of strict insulin titration afterwards. Nevertheless, the overall trend suggests a reduction in basal insulin dose by up to 17% and a decrease in prandial insulin dose by up to 30% in addition of GLP-1RA to insulin therapy, especially with long-acting forms [32].

Another study compared the effects of different antidiabetic treatments, including GLP-1RA, SGLT-2 inhibitors, and their combination, versus insulin. The study found that patients treated with GLP-1RA, SGLT-2i, or their combination showed greater improvements in various cardiovascular and metabolic markers compared to those treated with insulin alone after 12 months. Specifically, patients on GLP-1RA or combination therapy exhibited greater reductions in body mass index (BMI), central systolic blood pressure (SBP), and arterial stiffness as assessed by pulse wave velocity (PWV). They also showed improvement in various vascular markers such as endothelial glycocalyx thickness, global left ventricular strain, and myocardial work index. These effects were observed despite similar improvements in glycemic control measured by HbA1c reduction. The combination of GLP-1RA and SGLT-2i was found to produce additive cardiovascular benefits compared to either treatment with one agent alone [33].

While lifestyle interventions such as diet and exercise can normalize HbA1c, the benefits of GLP-1RA and SGLT2-i extend well beyond glycemic control and justify continuation of therapy. For example, in the EMPA-REG OUTCOME trial, patients randomized to receive empagliflozin experienced a significant reduction in cardiovascular death, all-cause mortality, and hospitalization for heart failure compared to placebo despite only modest differences in HbA1c between groups [22]. Importantly, the benefits emerged early and were consistent across all patient subgroups, including those with relatively well-controlled diabetes. These findings strongly suggest that SGLT2 inhibitors confer cardiovascular and renal protection through mechanisms independent of glycemic improvement. As such, discontinuing therapy solely based on normalized HbA1c may deprive patients, especially those with underlying cardiovascular or renal risk, of organ-protective effects that are both substantial and sustained. While specific guidelines on continuing therapy after HbA1c normalization are lacking, current evidence supports ongoing use of these agents due to their insulin-independent mechanisms of action.

### Transitioning from Insulin to GLP-1 Agonists

Transitioning from insulin to GLP-1 agonist medications presents several challenges. Once insulin therapy is initiated, patients are infrequently titrated off this regimen, even when newer medications with additional benefits become available. This is unsurprising since treatment of an insulin resistance with the administration of more insulin may eventually lead to a more insulin-resistant state. Furthermore, this treatment inertia is partly due to the absence of clear guidelines on how to reduce insulin doses when adding alternative glucose-lowering therapies, making it more likely that insulin therapy will continue uninterrupted. Hence, we propose a more proactive method to “de-insulinization” in conjunction with intensive lifestyle adjustment. The contemporary therapies, while associated with certain limitations—including difficulties with access, availability, and cost—permit easier dosing, exhibit fewer side effects (especially hypoglycemia), and facilitate weight loss, with ever-increasing evidence of salutary effects across a spectrum of medical disease states (e.g., obstructive sleep apnea, kidney disease, CHF).

### Identifying Candidates for Insulin Tapering

Careful patient selection can identify which patients have the highest likelihood of successfully transitioning off insulin to medications such as GLP-1 agonists. Independent retrospective studies by Kawata et al. (2014) and Bruinstroop et al. (2018) identified several factors that predict positive outcomes during this switch. Lower baseline insulin doses, shorter duration of diabetes, and lower HbA1c levels were associated with greater success in transitioning from insulin to a GLP-1 agonist [34, 35]. Patients with a baseline daily insulin dose of 19 units and a disease duration of 9 years were found to be the cut-off values using the area under the receiver operating characteristic (ROC) curve analysis. In these studies, insulin was discontinued, and patients were started on liraglutide at a low dose, which was subsequently titrated based on tolerance and need.

Beyond these predictors, additional patient characteristics can guide the decision to transition. One analysis of patients from insulin to an insulin/GLP-1RA fixed combination identified candidates that would benefit from such a switch. As mentioned, GLP-1 medications provide a cardiovascular benefit in patients at risk for cardiovascular disease. These agents are also well-known to induce weight loss, making them suitable for patients with obesity or those seeking weight reduction. Furthermore, patients experiencing frequent hypoglycemia, poor glycemic control on current insulin regimens, or those using complex treatment strategies—such as multiple daily injections—may benefit from a switch to a simplified regimen with combination basal insulin and GLP-1 agonists [36]. Several studies have studied the addition of GLP-1RA along with dose titration/discontinuation of insulin therapy. A study published by Rosenstock et al. found that 72% of subjects who initiated albiglutide along with basal insulin no longer needed prandial insulin or used it at a lower dose [37]. Another retrospective study from a VA primary care clinic found 6.3% of the study group discontinued insulin use when they initially started a GLP-1 agonist, and 14.6% of patients discontinued insulin use within the year follow-up [38].

### Risks of Insulin Tapering In Patients on High-Dose Insulin

Adding another antidiabetic medication to patients on high-dose insulin—including those using multiple daily injections, continuous subcutaneous insulin infusion, or automated insulin delivery systems—along with sulfonylureas or meglitinides increases the risk of hypoglycemia [39]. Thus, caution should be taken in monitoring for hypoglycemia when adding additional anti-diabetic medication. The ACCORD trial found that intensive glucose-lowering therapies were associated with increased all-cause mortality in patients with a history of cardiovascular disease, underscoring the importance of cautious titration [40].

To mitigate hypoglycemia risk, recommendations regarding initial insulin dose reduction vary based on the patient’s initial HbA1c level. Previous studies examining the addition of a GLP-1 agonist have suggested an insulin dose reduction of 20% in patients with hemoglobin A1c levels < 8.0%. For patients with higher HbA1c levels, no initial insulin dose reduction is typically needed [41–43]. If hypoglycemia is avoided after the first month, insulin doses may be titrated upward based on finger-stick glucose readings [43]. For more granular insulin adjustments, another study suggested reducing insulin by 50% for patients with HbA1c < 7.0% and by 25% for those with HbA1c between 7.1 and 8.0% [42]. It is important to note that these recommendations are largely based on clinical experience and observational practice.

## Clinical Strategies for Safe Insulin Deprescription

### GLP-1RA Initiation and Insulin De-escalation

Misriky et al. reported a case study illustrating a successful transition of an obese patient on high-dose insulin to weekly semaglutide [41]. The authors highlighted their stepwise approach to insulin reduction, which involved prompt weekly insulin dose decreases of 10–20% when preprandial or fasting glucose levels were consistently between 80 and 130 mg/dL. To ensure patient safety and reduce hypoglycemia risk, follow-up occurred every 4 weeks, either in clinic or by phone. Patients were encouraged to self-adjust insulin doses by 10–20% weekly, depending on glucose levels [41].

Continuous glucose monitoring (CGM) can be a valuable tool during these transitions. CGM allows for real-time tracking of glucose trends and facilitates safe medication adjustments. The American Diabetes Association (ADA) recommends that patients spend at least 70% of their CGM readings within the target range of 70–180 mg/dL (3.9–10.0 mmol/L), with less than 4% of readings below 70 mg/dL (time below range, TBR) [39]. Glucose readings under 54 mg/dL, classified as level 2 hypoglycemia, should be limited to less than 1% of the time, as these episodes are potentially dangerous. The ADA also advises that time above range should not exceed 25% [39].

### SGLT2-i Initiation and Insulin De-Escalation

In a retrospective cohort study by Champion et al., initiation of SGLT2-i—often in combination with metformin and/or a GLP-1 receptor agonist—led to significant reductions in both insulin dose and “overbasalization” rates. Overbasalization refers to increasing the basal dose of insulin after the fasting glucose goal has been achieved, in an attempt to lower prandial glucose. Among patients treated with metformin and insulin, the proportion of overbasalized patients decreased from 57 to 36%, with complete insulin discontinuation achieved in all cases. In the triple therapy cohort receiving metformin, SGLT2-i, and GLP-1RA, overbasalization declined from 62.5 to 17.5%, and 42.5% of patients were able to stop insulin entirely, reinforcing the additive benefits of combination therapy [44].

Additional evidence comes from a randomized controlled trial by Naing et al., which tested a structured insulin tapering protocol in patients with poorly controlled T2DM on multiple daily insulin injections. In the intervention group, prandial insulin was discontinued and basal insulin reduced to 80% of the home dose or converted to glargine at 40% of the total daily dose for patients on premixed regimens. Empagliflozin was initiated at 10–12.5 mg daily and titrated to 25 mg, alongside dulaglutide and metformin. After 16 weeks, the intervention group achieved a mean HbA1c reduction of 2.38%, with 40% of patients reaching an HbA1c < 7% and a mean reduction in insulin dose of 57.3 units per day. Improvements in blood pressure, LDL, and total cholesterol were also observed, and no episodes of diabetic ketoacidosis or severe hypoglycemia episodes occurred [45].

**Fig. 1:**
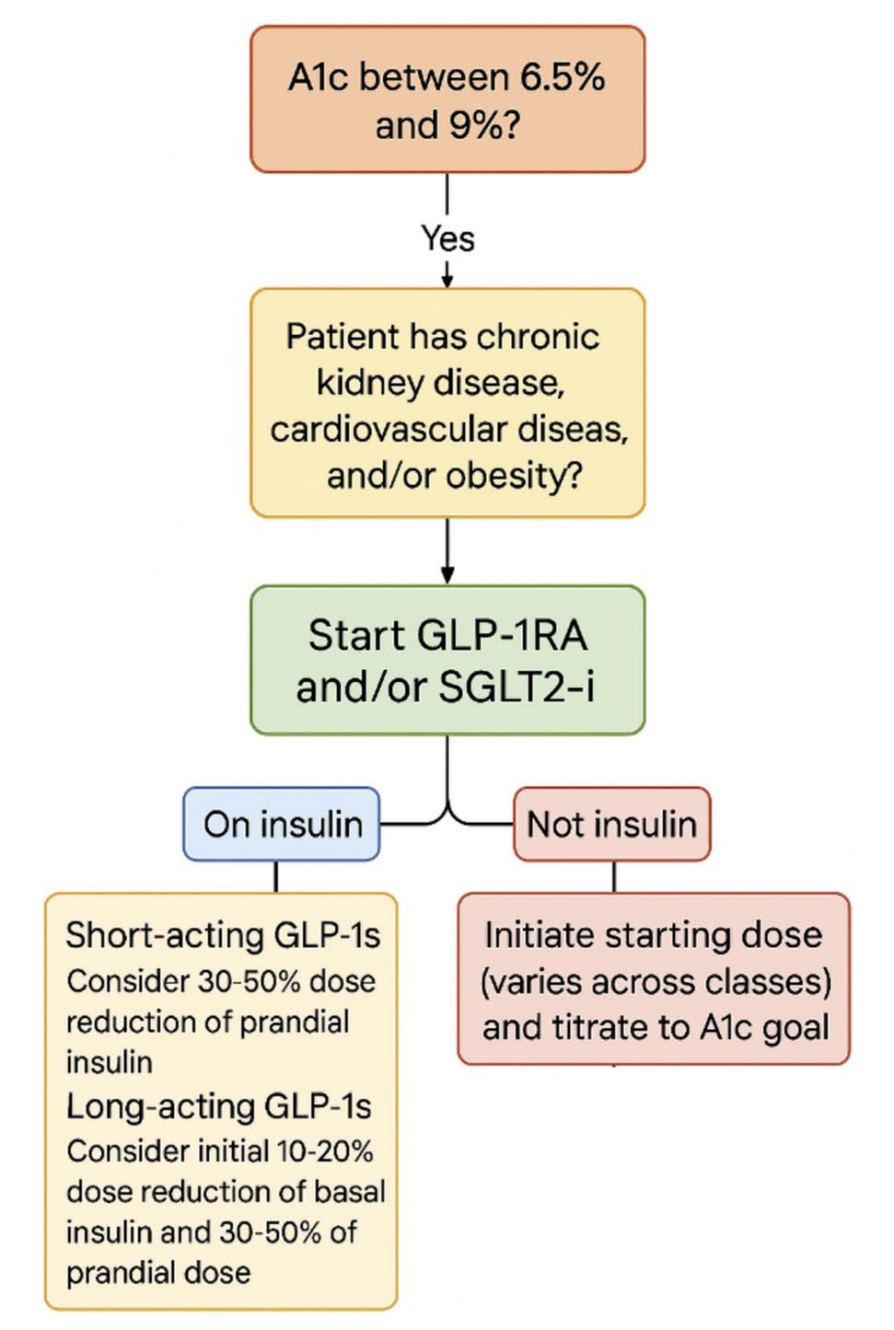
Treatment algorithm for initiating GLP-1RA and SGLT2-i based on A1c, comorbidities, and insulin use

Overall, SGLT2 inhibitors can be safely initiated with a 10–20% basal insulin reduction if HbA1c is < 8%, allowing prandial insulin tapering when fasting glucose is stable, especially when combined with GLP-1RA, though monitoring for hypoglycemia remains essential (Fig. 1).

## Limitations

### Adverse Effects

Although GLP1-RA help improve glycemic control, promote weight loss, and reduce cardiovascular events, there are several limitations to its use. Most common side effects include gastrointestinal symptoms such as nausea, vomiting, diarrhea, and constipation. These occur due to delayed gastric emptying and early satiety but typically peak with the initiation of the medication and decline over time with careful titration and monitoring. Counseling can be provided to patients so that they avoid eating while full to decrease their symptoms of nausea. Patients with a history of gastroparesis, inflammatory bowel disease, and pancreatitis should also avoid GLP 1 RA given the potential risk of worsening side effects [46]. Injection-site pruritus is another consideration and is typically seen with the longer-acting GLP-1RA. Overall, adverse effects account for approximately a 10% discontinuation rate of the medication. Major limitations for SGLT-2i initiation include limited use in patients with a history of recurrent genito-urinary tract infections, significant renal impairment, lower limb amputation, and euglycemic DKA. Patients starting on SGLT-2i with diabetes and heart failure should be monitored for hypoglycemia, fluid balance, and serum electrolytes. A drop in eGFR < 30% should also be anticipated with initiation in patients with CKD and should not be an indication for discontinuation [47].

One cross-sectional study from 2017 surveyed patients and physicians in the USA, Italy, France, Germany, Spain, and the UK on problems experienced with GLP-1RA. The most common reason for discontinuation globally was patient-related GI issues, with 64.4% reporting they caused malaise and 45.4% reporting emesis. Preference for oral medications over injections was also a common reason for discontinuation in this study [48].

### Cost

Cost emerges as another significant barrier, with GLP-1RA and SGLT-2i being more expensive than insulin. The monthly wholesale price for GLP-1RA can exceed $1000 prior to insurance, rebates, or other discounts. Cost was noted to be a significant factor for patients in the USA compared to other EU countries, with 48.6% of patients reporting discontinuation due to cost in the USA vs. 9.5% in the EU [48]. Given the demand for these agents, there have been several supply shortages leading to access issues and an increase in cost. In 2022, semaglutide, dulaglutide, and tirzepatide were all listed as “currently in shortage” per the FDA. The tirzepatide shortage has since been resolved, but semaglutide, liraglutide, and dulaglutide are all still in shortage on the FDA drug shortage database [49]. Interestingly, the shortage has not affected product doses equally, with some doses more available than others. This has led patients to obtain GLP-1RA through social media websites and commercial companies that may be compounded from an unknown source, posing other health risks. Physicians are working to create alternative dosing options for these medications to help maintain the total desired weekly or monthly dose. A few articles have outlined strategies for missed doses, extended interval dosing, and interchanging between GLP1-RA [50].

### Tapering off GLP1-RA

In the STEP 1 extension, a cohort of 1961 adults with body mass index ≥ 30 (or ≥ 27 with ≥ 1 weight‐related co‐morbidity) after 68 weeks of treatment with once-weekly semaglutide 2.4 mg and lifestyle interventions (diet counseling every 4 weeks and 150 min of physical activity weekly) were studied. At the end of week 120, the participants who received semaglutide regained 11.6 percentage points of lost weight compared to 1.9 percentage points in the placebo group. The participants that had the most weight regained after withdrawal had lost 20% or more of their baseline body weight during treatment. Cardiometabolic factors including systolic and diastolic blood pressure, CRP, and lipid levels that had improved from weeks 0 to 68 were also seen to revert towards baseline after withdrawal of semaglutide. In a prior trial STEP 4, patients were observed off semaglutide after a 20-week treatment period but continued with lifestyle intervention support. The trajectory of weight gain in this study was relatively smaller than STEP 1, possibly due to the smaller amount of weight loss in this group but also due to continued lifestyle intervention support after medical withdrawal as mentioned above [51].

This rebound phenomenon on cessation is somewhat discouraging and suggests that long-term effects may be expected. However, as with any relatively novel therapy, possible consequences of indefinite therapy will only be revealed with time. Further work is needed to see if short-term GLP1-RA use in conjunction with aggressive lifestyle therapy can jumpstart “de-insulinization” similar to a short-term course of varenicline or nicotine replacement therapy for tobacco users.

## Future Directions

Although not identical, food addiction and substance use disorder exhibit similar neurological, physiological, and behavioral abnormalities. Patients undergoing treatment for smoking cessation have been observed to consume significantly less alcohol after 12 weeks of treatment with dulaglutide compared to those receiving a placebo, irrespective of their smoking status. The results suggest a positive effect of GLP-1RA on reducing alcohol intake in humans. This study highlights the need for further research of GLP-1RA in treating alcohol use disorder and substance use disorder [52].

GLP-1 receptor agonists may also influence the addictive properties of cocaine, amphetamine, opioids, and nicotine. In the case of cocaine, GLP-1 receptor agonists have demonstrated the ability to suppress behaviors associated with cocaine use in rodents, such as conditioned place preference, self-administration, and hyperactivity. Regarding amphetamine, GLP-1 receptor activation has been observed to reduce amphetamine-induced conditioned place preference and locomotor activity in animal models. Studies on opioids have yielded mixed results, with some reporting a decrease in opioid self-administration with GLP-1 receptor agonist treatment, while others have found no significant effects. Possible mechanisms of action of GLP-1RA were proposed. GLP-1 receptor stimulation could promote satiety or avoidance of aversive drug effects through habenula-dependent mechanisms [53].

Furthermore, the effects of GLP-1 receptor agonists on nutrient intake could contribute to their impact on substance use, but evidence suggests that their effects on reward and reinforcement are independent of these factors. GLP-1 receptors in the brain are heterogeneous and may exert different effects depending on the region and second messenger pathways involved, suggesting the existence of different functional subtypes. These variations in receptor expression and signaling may contribute to differences in the efficacy and tolerability of different GLP-1 receptor agonists in substance use disorder treatment [53].

Another role for GLP-1RA is in the treatment of eating disorders. Animal research has revealed both the central and peripheral effects of GLP-1RA in modulating brain-gut signals, appetite circuits, and cravings through dopaminergic and serotonin pathways. Several prospective studies have been conducted in patients with binge eating disorders and psychiatric disorders leading to compulsive eating. Semiglutide, liraglutide, and dulaglutide have all been seen to reduce binge eating scores in separate case reports [54].

## Conclusions

In conclusion, both GLP-1RA and SGLT2-i exert a myriad of cardiovascular benefits for patients with diabetes, obesity, heart failure, and chronic kidney disease. These agents can be carefully initiated and titrated by cardiologists, with limitations and adverse effects under consideration. Further work is needed to explore consequences of long-term use of these medications, ideal insulin deprescription protocols, and strategies to alternatively dose these medications around ongoing shortages.

## Data Availability

No new data were generated or analyzed in this study; therefore, data sharing is not applicable to this article.

## Code Availability

No custom code was developed for this review article.
